# The Role of Tricarboxylic Acid Cycle Metabolites in Viral Infections

**DOI:** 10.3389/fcimb.2021.725043

**Published:** 2021-09-14

**Authors:** Francisco Javier Sánchez-García, Celia Angélica Pérez-Hernández, Miguel Rodríguez-Murillo, María Maximina Bertha Moreno-Altamirano

**Affiliations:** Laboratorio de Inmunorregulación, Departamento de Inmunología, Escuela Nacional de Ciencias Biológicas, Instituto Politécnico Nacional, Mexico City, Mexico

**Keywords:** viruses, host cell metabolism, metabolic reprogramming, mitochondria, tricarboxylic acid cycle

## Abstract

Host cell metabolism is essential for the viral replication cycle and, therefore, for productive infection. Energy (ATP) is required for the receptor-mediated attachment of viral particles to susceptible cells and for their entry into the cytoplasm. Host cells must synthesize an array of biomolecules and engage in intracellular trafficking processes to enable viruses to complete their replication cycle. The tricarboxylic acid (TCA) cycle has a key role in ATP production as well as in the synthesis of the biomolecules needed for viral replication. The final assembly and budding process of enveloped viruses, for instance, require lipids, and the TCA cycle provides the precursor (citrate) for fatty acid synthesis (FAS). Viral infections may induce host inflammation and TCA cycle metabolic intermediates participate in this process, notably citrate and succinate. On the other hand, viral infections may promote the synthesis of itaconate from TCA cis-aconitate. Itaconate harbors anti-inflammatory, anti-oxidant, and anti-microbial properties. Fumarate is another TCA cycle intermediate with immunoregulatory properties, and its derivatives such as dimethyl fumarate (DMF) are therapeutic candidates for the contention of virus-induced hyper-inflammation and oxidative stress. The TCA cycle is at the core of viral infection and replication as well as viral pathogenesis and anti-viral immunity. This review highlights the role of the TCA cycle in viral infections and explores recent advances in the fast-moving field of virometabolism.

## Overview of the TCA Cycle

Comprehensive reviews on metabolic pathways, including the TCA cycle and their role beyond metabolism, have recently been published (Claus and Liebert, 2014; O´Neill et al., 2016; Williams and O´Neill, 2018; Martínez-Reyes and Chandel, 2020; Choi et al., 2021). In brief, the TCA cycle (also known as Krebs cycle or citric acid cycle) takes place within mitochondria and initiates with a reaction catalyzed by the enzyme citrate synthase, leading to the synthesis of citrate from the condensation of oxaloacetate with acetyl-coenzyme-A. The latter is produced outside the mitochondria from the catabolism of carbohydrates, proteins, and fatty acids, as well as from the breakdown of the citrate exported from mitochondria to the cytosol. From citrate, the TCA cycle proceeds through eight enzymatic reactions whereby citrate is converted to cis-aconitate, cis-aconitate to isocitrate, isocitrate to α-ketoglutarate, α-ketoglutarate to succinyl-CoA, which is then converted to succinate, succinate to fumarate, fumarate to malate and finally, malate to oxaloacetate which upon condensation with acetyl-coenzyme-A initiates a new TCA cycle.

The TCA cycle is linked to the electron transport chain (ETC) at the succinate dehydrogenase (SDH, mitochondrial respiratory complex II). The TCA cycle produces NADH (nicotinamide adenine dinucleotide, in its reduced form) and FADH2 (flavin adenine dinucleotide, in its reduced form), which are oxidized by the mitochondrial ETC for the synthesis of ATP.

Metabolic diversions from the TCA cycle may take place and in this way citrate can exit from the TCA cycle and be exported to the cytoplasm in exchange for malate by the family 25 member 1 (Slc25a1) solute carrier.

**Figure 1:**
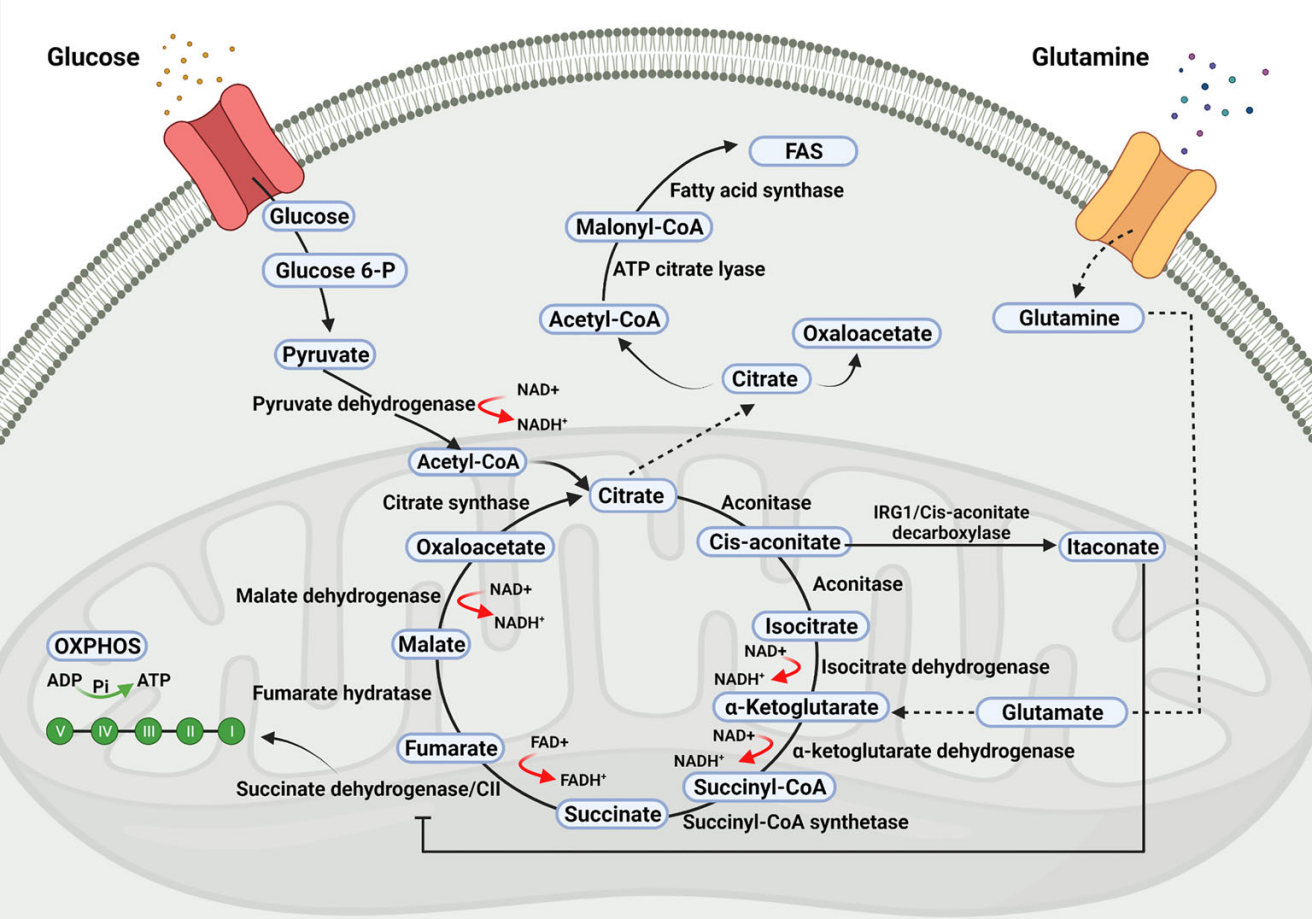
Tricarboxylic acid (TCA) cycle. The TCA cycle takes place within the mitochondria and it is the source of precursors for the synthesis of biomolecules required for viral replication as well as of metabolites with signalling properties which may influence the outcome of viral infections.

Cytosolic citrate is converted into oxaloacetate and acetyl-CoA, which is converted into malonyl-coenzyme-A to be incorporated into cholesterol or fatty acids (Williams and O´Neill, 2018). Likewise, cis-aconitate can be diverted from the synthesis of iso-citrate to produce itaconate instead, by means of the cis-aconitate decarboxylase enzyme, the product of the immune-responsive gene 1 (Irg1), latter renamed as Acod1 (Williams and O´Neill, 2018). Itaconate is an inhibitor of SDH, inhibiting the conversion of succinate into fumarate (**Figure 1**).

## TCA Cycle and Virus Replication Cycle

The cross-talk between host cell metabolism and viruses may be approached from different but not excluding perspectives: 1) metabolism and virus replication cycle 2) metabolism and anti-viral immunity 3) virus control of immune cells metabolism for the subversion of anti-viral immune responses 4) metabolism and inhibition of viral replication, and 5) metabolite-dependent viral pathogenicity (Lewis and Scott, 1962; Landini, 1984; Maynard et al., 2010; Yu et al., 2011; Sánchez and Lagunoff, 2015; Netea et al., 2016; Eisenreich et al., 2019; Mayer et al., 2019; Moreno-Altamirano et al., 2019; Olagnier et al., 2020).

Pioneering work analyzing the role of host cell metabolism on viral replication goes back to the early 1950s when Ackermann et al. first observed that *ex vivo* influenza A virus infection of mice tissues was impaired by the addition of chemical inhibitors of TCA cycle enzymes (Ackermann et al., 1951).

Viruses require that their host cells synthesize lipids, proteins and nucleic acids to ensure a viral progeny, and that they produce energy to drive viral assembly and release (Hui and Nayak, 2001; Claus and Liebert, 2014). Fatty acid synthesis requires glycolysis, pentose phosphate pathway, and TCA cycle metabolic intermediates. TCA cycle-derived citrate may be exported out of the mitochondria towards the cytosol where it is converted to acetyl-CoA by the ATP citrate lyase enzyme and then to malonyl-CoA by the acetyl-CoA carboxylase enzyme, from malonyl-CoA, the elongation of straight-chain fatty acids proceeds sequentially by means of the fatty acid synthase, in an NADPH-dependent reaction, up to the formation of palmitate (C16); further elongation and the insertion of double bonds require other enzyme systems. The synthesis of branched-chain fatty acid requires branched-chain amino acids as substrates, and triacylglycerols and phospholipids are synthesized from the condensation of fatty acids with glycolysis-derived glycerol (O’Neill et al., 2016).

Glutamine is converted to glutamate, which is incorporated into the TCA cycle as α-ketoglutarate, providing the precursors for aspartic acid, asparagine, glutamic acid, and proline, as well as citrate. The cell uptake of glutamine allows the flux of glucose to the pentose-phosphate pathway and, amino acid, nucleotide, and fatty acids biosynthesis (Boroughs and DeBerardinis, 2015).

Viral replication is highly dependent on glucose, glutamine, and the TCA cycle (Mayer et al., 2019). Therefore, how viruses might take control of those metabolic substrates and subvert the TCA cycle to support their replication cycle seems to be a fundamental question. In this regard, the herpes simplex virus-1-infected cells may shift glucose metabolism to nucleotide synthesis instead of glycolysis or the TCA cycle, whereas glutamine feeds the TCA cycle, through the synthesis of α-ketoglutarate (Vastag et al., 2011; Mayer et al., 2019). Other viruses induce glycolysis or are benefited from a glycolytic cell host, these include dengue virus (DENV) (Fontaine et al., 2015), hepatitis C virus (HCV) (Ramiere et al., 2014), human immunodeficiency virus (HIV) (Palmer et al., 2014), human adenovirus-2 (HAdV-2) (Guissoni et al., 2018), Zika virus (ZIKV) (Singh et al., 2020), and SARS-CoV-2 (Bojkova et al., 2020).

Human adenovirus HAdV-2 infection increases glucose consumption and glycolysis, as well as glutaminolysis, which replenish TCA cycle, whereas the TCA cycle itself undergoes a 1.5 fold increase in the production of metabolic intermediates (Carinhas et al., 2017). Proteomic analysis of cells infected with HAdV-2 showed the up-regulation of glycolysis and TCA cycle enzymes, along with those of other metabolic pathways (Guissoni et al., 2018). Zika virus infection up-regulates several genes involved in glycolysis, including the glucose transporter 1 (GLUT1), and the monocarboxylate transporter MCT4, which expulse lactate outside cells (Singh et al., 2020). Moreover, inhibition of glycolysis impairs replication of herpes simplex virus (Courtney et al., 1973; Mayer et al., 2019), and SARS-CoV-2 (Bojkova et al., 2020).

In addition to their role as a source of energy and biomolecule precursors for virus assembly, TCA metabolites may serve signaling roles (Williams and O’Neill, 2018; Hooftman et al., 2020), all of which open new avenues for the understanding of host cell-virus interaction, and the view of cell metabolism as a possible target for anti-viral intervention.

## TCA Intermediate Metabolites With Known Signaling Properties

The TCA cycle metabolic intermediates, citrate, succinate, and fumarate, are known to harbor signaling properties (McGettrick and O’Neill, 2013; Mills and O’Neill, 2014; Williams and O’Neill, 2018). In addition, itaconate, a derivative from TCA cycle cis-aconitate is also a signaling molecule (O’Neill and Artyomov, 2019; Hooftman et al., 2020).

## Citrate and Its Role in Viral Infection

The mitochondrial citrate carrier exports citrate to the cytosol to be converted into acetyl-CoA and oxaloacetate. A key role of acetyl-CoA in the inflammatory process is the acetylation of histones, leading to the increased synthesis of IL-1β, TNF-α, and IFN-γ (Ashbrook et al., 2015; Mills et al., 2017; Williams and O´Neill, 2018).

Citrate links carbohydrate and fatty acid metabolism. Fatty acid synthesis is necessary for virus replication since it facilitates viral entry, replication, and egress from host cells (Chukkapalli et al., 2012). Most viruses replicate in specific membranous compartments, which differ from virus to virus. Flaviviruses, for instance, actively regulate lipid biosynthesis to establish their replication sites (Rothwell et al., 2009; Heaton et al., 2010; Chukkapalli et al., 2012). The dependence on fatty acid synthesis has been studied in several other viruses including dengue, West Nile, and hepatitis C (Heaton and Randall, 2011; Alvisi et al., 2011; Zhang et al., 2016; Starting and van Kuppeveld, 2017).

Citrate-derived acetyl-CoA has a role in eukaryotic histone acetylation that can be regulated by viral infections (Lu et al., 2003; Hancock et al., 2010). In addition, acetyl-CoA participates in the acetylation of non-histone proteins (Choudhary et al., 2009; Christensen et al., 2019). Protein acetylation is a prevalent lysine post-transcriptional modification that regulates epigenetics (histone acetylation) and protein function (non-histone acetylation). As a way of an example, NIH3T3 cells infected with Moloney murine leukemia virus and treated with histone deacetylase (HDAC) inhibitors increase the expression of unintegrated retroviral DNA, without increasing the amount of integrated DNA. First supporting the notion that unintegrated DNAs are functionally associated with nucleosomes and secondly that histone acetylation increases the stability of unintegrated DNA, thus increasing the viral template availability for transcription (Wang et al., 2016).

On the other hand, some viruses might regulate the acetyation of several host proteins contributing to host antiviral response as well as to viral replication (Murray et al., 2018). In this way, the interferon inducible protein IFI16 is acetylated within its nuclear localization motif, determining its cellular location and therefore its ability to initiate anti viral DNA innate immune response (Li et al., 2012).

**Figure 2:**
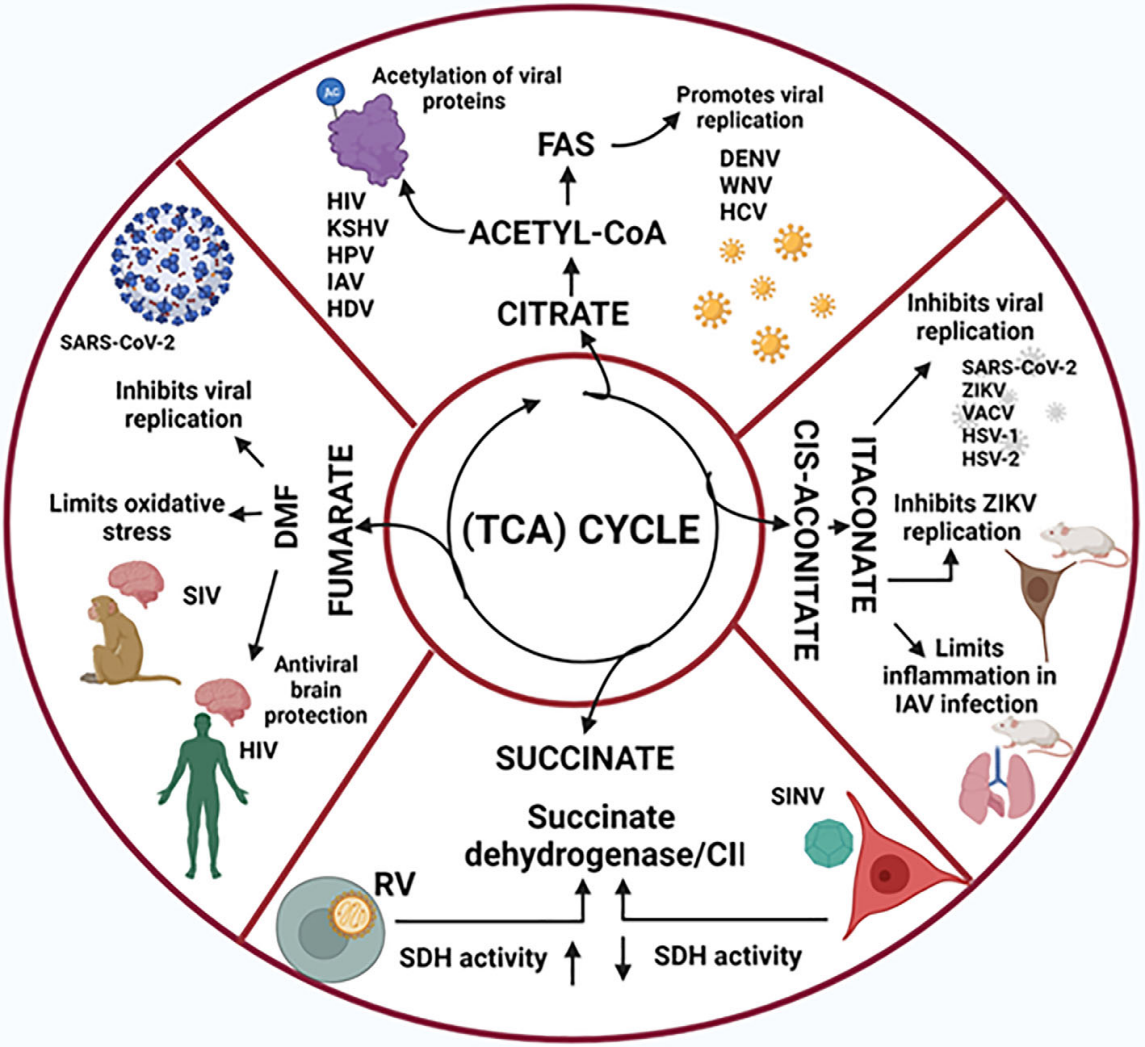
Tricarboxylic acid (TCA) cycle-derived metabolites and their role in viral infections. The TCA cyce intermediate metabolites citrate, succinate and fumarate play key roles in vral replication and in the virus-induced inflammation process. In addition, itaconate, which is synthesized from the TCA cis-aconitate, is also endowed with anti-inflammatory and anti-viral properties.

Several viral proteins are the targets of acetylation, a process that regulates virus cellular location, viral transcriptional activities, and virulence, such proteins include the HIV Tat protein, the Kaposi’s sarcoma-associated herpesvirus latency-associated nuclear antigen, the human papillomavirus E2 protein, the H1N1 influenza virus NS1 protein, and the hepatitis D virus and influenza virus nucleocapsids (Ott et al., 1999; Mu et al., 2004; Lu et al., 2006; Quinlan et al., 2013; Hatakeyama et al., 2018; Ma et al., 2020) (**Figure 2**). Acetylome analyzes during *in vitro* infection with human cytomegalovirus have showed that amongst the proteins that display increased acetylation are the pyruvate dehydrogenase (E1), dihydrolipoamide S-acetyltransferase (E2), and the dihydrolipoamide dehydrogenase (E3) components of the pyruvate dehydrogenase complex, which catalyzes the oxidation of pyruvate to acetyl-CoA and CO_2_ and interestingly, the dynamic translocation of the mitochondrial pyruvate dehidrogenase complex to the nucleus ensures the nuclear production of acetyl-CoA necessary for histone acetylation and epigenetic regulation (Murray et al., 2018).

Specific viral proteins might regulate the TCA cycle. The vaccinia virus for instance, encodes a viral homolog of the epidermal growth factor called viral growth factor which binds to the epithelial growth factor receptor, activates MAPK signaling and phosphorylates the signal transducer and activator of transcription 3 (STAT3) at the non-canonical site serine727. In this way vaccinia virus induces a 3.3-fold increase in the production of citrate and other TCA cycle metabolic intermediates at 8 h post infection (Pant et al., 2021), thus contributing to fatty acid synthesis and protein acetylation. Since protein acetylation regulates enzyme activity, chromatin structure, protein localization, and protein-protein interaction (Murray et al., 2018), the function of specific acetylated proteins and its impact on viral replication cycle remains to be analyzed.

## Itaconate and Its Role in Viral Infection

By using integrated high throughput transcriptional-metabolic profiling during murine macrophage M1 polarization, Jha et al. (Jha et al., 2015) analyzed a TCA cycle break at isocitrate dehydrogenase, the metabolic step that converts isocitrate to α-ketoglutarate, and demonstrated that in the TCA broken cycle, citric acid is redirected to itaconic acid synthesis, by the up-regulation of the immunoresponsive gene 1 (Irg-1, currently known as Acod-1), which encodes the cis-aconitate decarboxylase enzyme that catalyzes the conversion of cis-aconitate to itaconate (Michelucci et al., 2013; Németh et al., 2016).

Itaconate inhibits SDH, thus regulating succinate levels, mitochondrial respiration and the production of pro-inflammatory cytokines (Cordes et al., 2016; Lampropoulou et al., 2016; Cordes and Metallo, 2021). In addition, itaconate alkylates cysteine residues in the KEAP1 (Kelch-like ECH-associated protein 1) protein, which normally associates with Nrf2 (Nuclear factor erythroid 2 (NF-E2)-related factor 2), promoting its degradation. KEAP1 alkylation prevents Nrf2 degradation, allowing its accumulation, nuclear translocation, and the transcriptional activation of anti-oxidant and anti-inflammatory programs (Hayes and Dinkova-Kostova, 2014; Mills et al., 2018). An extracellular source of itaconate or its derivatives may regulate succinate levels, type I interferons, and reduce the expression of pro-IL-1β, IL-6, IL-12, and iNOS in macrophages (Lampropoulou et al., 2016; Swain et al., 2020). Itaconate may modify target proteins at cysteine residues, contributing to its role as an immunomodulator (Hooftman and O´Neil, 2019). On the other hand, itaconate induces electrophilic stress and inhibition of the IκBζ-mediated inflammatory program (Bambouskova et al., 2018). IκBζ up-regulates the expression of secondary response genes that enhance the TLR/IL-1R signalling pathways leading to cytokine production (Yamamoto et al., 2004; Ohto-Ozaki et al., 2020).

The itaconate derivative 4-octyl-itaconate (4-OI) limits the SARS-CoV-2 infection- associated host inflammatory response while inhibiting SARS- CoV-2 replication, independently of type I interferon (IFN) signaling (Olagnier et al., 2020), 4-OI also inhibits the replication of herpes simplex virus-1 and-2 (HSV-1 and HSV-2), vaccinia virus (VACV), and Zika virus (Olagnier et al., 2020).

Infection of mice with influenza A virus induces the expression of Acod-1 and the synthesis of itaconate which limits pulmonary inflammation and disease severity, whereas treatment with exogenous itaconate or its derivative dimethyl-itaconate reduces IFN responses and down-modulate pro-inflammatory cytokines, also limiting inflammation (Sohail et al., 2021). On the other hand, the itaconate derivative 4-octyl-itaconate suppresses influenza A virus replication in PBMC (Sohail et al., 2021). Thus itaconate and its derivatives limit inflammation and related pathologies, and suppresses viral replication.

Previously, using a murine model of ZIKV infection, Daniels et al. had shown that ZIKV activates a signaling pathway involving a the receptor interacting protein kinase-3 (RIPK3), leading to the up-regulation of Acod-1 and the production of itaconate, which suppresses the replication of ZIKV in neurons (Daniels et al., 2019).

It was recently shown that the expression of the Nrf2 gene which regulates the transcription of the glutathione and thioredoxin anti-oxidant systems, detoxification, NADPH regeneration, and heme metabolism (Tonelli et al., 2018) is suppressed in biopsies obtained from COVID-19 patients (Olagnier et al., 2020). In addition, itaconate induces electrophilic stress and inhibition of the IκBζ-mediated inflammatory program (Bambouskova et al., 2018). IκBζ up-regulates the expression of secondary response genes that enhance the TLR/IL-1R signalling pathways leading to cytokine production (Yamamoto et al., 2004; Ohto-Ozaki et al., 2020).

In addition to its anti-oxidant and anti-inflammatory roles, Nrf2 can also regulate the sensing of viral DNA in the cytoplasm and thus the release of antiviral type I IFNs, by decreasing STING mRNA stability, linking itaconate synthesis (or cell treatment with the itaconate derivative 4-O-I) with antiviral cytosolic DNA sensing (Olagnier et al., 2018) (**Figure 2**).

## Succinate and Its Role in Viral Infection

Lipopolysaccharide (LPS) activation of macrophages shifts cell metabolism to glycolytic ATP production, releasing mitochondria from ATP production enabling the increase of mitochondrial membrane potential and succinate synthesis, which oxidation by SDH, leads macrophages to an inflamatory state. The stimulation of dendritic cells and macrophages with LPS drives the succinylation of glyceraldehyde 3-phosphate dehydrogenase (GAPDH), lactate dehydrogenase (LDH), malate dehydrogenase (MDH) and glutamate carrier 1 (Tannahill et al., 2013; Mills et al., 2016).

Succinate is a pro-inflammatory metabolite as well as a competitive inhibitor of various α-ketoglutarate-dependent dioxygenases, such as histone demethylases and prolyl hydroxylases. The succinate receptor SUCNR1/GPR91, might signal in synergy with Toll-like receptor 3 and Toll-like receptor 7 to increase the synthesis of pro-inflammatory cytokines, whereas the inhibition of histone demethylases and prolyl hydroxylases contributes to the epigenetic regulation (Rubic et al., 2008; Xiao et al., 2012).

Whether virus-induced glycolysis is associated with other traits of cell activation-induced glycolysis, such as succinate metabolism, requires further analysis. However, there are some instances in which SDH activity is altered during viral infection (Escoll et al., 2019). Infection of three different human cell lines with rubella virus strongly increases SDH activity, along with a slight increase of respiratory complex III activity and decreased activity of complex IV (Claus et al., 2013; Escoll et al., 2019), whereas infection of a mouse neuroblastoma cell line with the Sindbis virus decreases SDH activity, along with respiratory complex I (Silva da Costa et al., 2012; Escoll et al., 2019).

While the role of succinate as a signal in the communication between host cells and pathogenic bacteria is being unveiled (Rosenberg et al., 2021), the role of succinate on viral infections is less studied (**Figure 2**).

## Fumarate and Its Role in Viral Infection

Fumarate is the product of the oxidation of succinate, by the succinate dehydrogenase enzyme. Fumarate and its derivatives monomethyl fumarate (MMF) and dimethyl fumarate (DMF) are potent immunomodulators and anti-oxidants that activate Nrf2 (Cecchini, 2003; Wilms et al., 2010; Cross et al., 2011; Linker et al., 2011). DMF inhibits maturation of dendritic cells (DCs) (Peng et al., 2012) and drives the production of IL-10, IL-12 and IL-23 by DCs thus down-regulating pathogenic T lymphocytes (Schlöder et al., 2017), DMF also inhibits Th1 to Th2 lymphocyte transition, pro-inflammatory cytokine signaling, the nuclear translocation of the transcription factor NF-kB, and the expression of cell adhesion molecules in lymphocytes and endothelial cells (Rubant et al., 2008; Gillard et al., 2015; McGuire et al., 2016; Wu et al., 2017).

DMF has been approved for the treatment of multiple sclerosis and might also protect against systemic and central nervous system complications in HIV infection (Gill and Kolson, 2013). In a study on rhesus macaques infected with simian immunodeficiency virus, treatment with DMF limited the oxidative stress throughout the brain (Garcia-Mesa et al., 2021). Olagnier et al. demonstrated that DMF inhibits SARS-CoV-2 replication and the expression of associated inflammatory genes, thus proposing that DMF could be repurposed as a small molecule inhibitor of SARS-CoV-2 replication and inflammation-associated pathology in COVID-19 patients (Olagnier et al., 2020; Timpani and Rybalka, 2021) (**Figure 2**). It is likely that inflammatory processes associated with other viral infections could also be treated with DMF.

As a note of caution, the use of DMF induces lymphopenia in about 3% of the patients so treated for relapsing-remitting multiple sclerosis, and at least one case of herpes simplex encephalitis associated with DMF treatment has been reported (Perini et al., 2018).

## Concluding Remarks

In addition to their role in biosynthesis and energy production which are critial for viral infections, the TCA cycle metabolites citrate, succinate and fumarate, as well as itaconate, which is synthesized from the TCA cycle cis-aconitate, play key roles in the pro-inflammatory/anti-inflammatory homeostasis, contributing to both anti-viral immune response and virus-induced inflammation and associated pathologies. The study of these metabolites in the context of viral infections offers the opportunity to understand better the host cell-virus interaction and to develop new pathophysiological assessment tools and anti-viral therapeutics.

The use of serum concentrations of TCA metabolites as biomarkers of infection and pathology is a promising approach. The serum concentrations of succinate, α-ketoglutarate, fumarate and itaconate, for instance, have been used for the clinical follow up of sepsis (Beloborodova et al., 2019) and more recently the serum metabolome of COVID-19 patients has informed that succinate levels are higher and citrate levels are lower, as compared with healthy controls, suggesting that these two metabolites along with D-fructose and 2-palmitoyl-glycerol are of value in the pathogenesis and diagnosis of COVID-19 (Shi et al., 2021).

The specific role of TCA cycle metabolites on the infection process with particular virus species is being unveiled, and comparative analyses will advance our understanding of virus biology as well as virus-host cell interaction. Finally, the use of TCA metabolites and their derivatives as anti-inflammatory, anti-oxidant, and anti-viral molecules, are paving the way to metabolism-targeted anti-viral therapeutic intervention. We foresee a rapid development in this area.

## Author Contributions

FS-G: Conceived the review, wrote the manuscript and approved final version. CP-H: Contributed with writing, review and approved final version MR-M: Contributed with figures design, review and approved final version MM-A: Conceived the review, wrote the manuscript and approved final version. All authors contributed to the article and approved the submitted version.

## Conflict of Interest

The authors declare that the research was conducted in the absence of any commercial or financial relationships that could be construed as a potential conflict of interest.

## Publisher’s Note

All claims expressed in this article are solely those of the authors and do not necessarily represent those of their affiliated organizations, or those of the publisher, the editors and the reviewers. Any product that may be evaluated in this article, or claim that may be made by its manufacturer, is not guaranteed or endorsed by the publisher.
